# Supplementary material for: Subsurface microbial community structure shifts along the geological features of the Central American Volcanic Arc
Source: PLoS One. 2024 Nov 13;19(11):e0308756. doi: 10.1371/journal.pone.0308756 (PMC11560019; doi:10.1371/journal.pone.0308756)

# Supplementary Information

## Supplementary Material and Methods

**Geological context.** The Cocos oceanic plate subducts beneath the Caribbean plate at a rate of 8-9 cm/yr, the Nazca oceanic plate converges eastward at 6 cm/yr relative to Northwestern South America (NWSA), and the Caribbean plate moves at 1-2 cm/yr to the E-SE relative to NWSA^1^. The Panama block moves independently from both the Nazca and Caribbean Plates and is bounded to the north by subduction of the Caribbean Plate along a series of fold and- thrust belts called the Northern Panama Thrust Belt^2^. Recent studies performed in southern Costa Rica and Panama suggest that the slab in the northwestern flanks of the Cocos Ridge dip gently (∼20°) for approximately 100 km from the trench and abruptly steepen (up to 80°) farther downdip^1^. There is (i) a significant difference in obliquity of the convergent margin between Costa Rica and Panama, (ii) a decreasing north/south gradient in thickness of the crust of the lower plate, and (iii) a potential increasing west/east gradient in slab dip and slab depth in accordance with distance from the subduction zone. All these changes contribute to variations in the volcanism along CAVA, with an interruption in active volcanism in Panama corresponding to a slab window^1,2^.

**Supplementary References**

1. Bekaert, D. V. et al. High 3He/4He in central Panama reveals a distal connection to the Galápagos plume. PNAS 118, e2110997118 (2021).

2. Guillermo, A., Alvarado, & Cárdenes. Geology, Tectonics, and Geomorphology of Costa Rica: A Natural History Approach. in (2017).

3. Giggenbach, W. F. Geothermal solute equilibria. Derivation of Na-K-Mg-Ca geoindicators. Geochimica et Cosmochimica Acta 52, 2749–2765 (1988).

## Supplementary Tables

**Supplementary Table S1**. Location and physico-chemical parameters of the sampled sites. Sample IDs that have numbers starting with 17 are sites where 16S rRNA gene amplicon data was published in Fullerton et al., 2021.

| **Code** | **Site name** | **Sample iD** | **Latitude** | **Longitude** | **Altitude** | **Region** | **Temperature** | **Salinity** | **pH** | **DIssolved Oxygen** | **³HE/^4^He** | **Cl^-^** | **SO_4_^2-^** | **DIC** | **Na^+^** | **K^+^** | **Mg^2+^** | **Ca^2+^** | **Rock type** |
| --- | --- | --- | --- | --- | --- | --- | --- | --- | --- | --- | --- | --- | --- | --- | --- | --- | --- | --- | --- |
|  |  |  | (°N) | (°E) | (m asl) |  | (°C) | (‰) |  | (%) | (Rc/Ra) | (ppm) | (ppm) | (ppm) | (ppm) | (ppm) | (ppm) | (ppm) |  |
| BC | Los Bajos the Corera | BC180410 | 8.806037 | -79.790968 | 18 | Panama slab window | 31.8 | 23.87 | 7.5 | 51.0 | 4.0 | 165.54 | 0.42 | 56.85 | 56.46 | 1.52 | 2.09 | 0.27 | Rhyolite |
| BQ | Borinquen | BQ170218 | 10.810883 | -85.413707 | 535 | Active volcanic arc | 88.9 | 5.73 | 2.1 | 9.9 | 5.1 | 0.35 | 18.72 | NA | 0.73 | 0.19 | 2.23 | 3.92 | Andesite |
| BR1 | Blue River 2 | BR170218_2 | 10.898370 | -85.328530 | 437 | Active volcanic arc | 59.0 | 3.25 | 6.2 | 7.0 | 6.5 | 29.03 | 4.23 | 9.56 | 9.28 | 3.03 | 11.56 | 5.85 | Andesite |
| BR2 | Blue River 1 | BR170218_1 | 10.898370 | -85.328530 | 434 | Active volcanic arc | 53.0 | 3.25 | 6.2 | 41.0 | 6.5 | 16.75 | 3.72 | 9.31 | 5.52 | 1.75 | 6.75 | 5.45 | Andesite |
| BS | Bajo Mendez Spring | BS180407 | 8.666450 | -82.349100 | 360 | Cordillera Talamanca | 40.9 | 3.10 | 9.1 | 20.0 | 5.7 | NA | NA | NA | 8.53 | 0.08 | 0.07 | 5.24 | Andesite |
| BW | Bajo Mendez Well | BW180407 | 8.665810 | -82.348670 | 362 | Cordillera Talamanca | 43.2 | 3.13 | 9.1 | 3.0 | NA | 21.54 | 1.29 | 0.36 | 17.10 | 0.08 | 0.07 | 8.41 | Andesite |
| CH | Chiguiri Abajo | CH180410 | 8.705080 | -80.269190 | 216 | Panama slab window | 31.1 | 15.30 | 7.0 | 41.0 | 6.4 | 74.96 | 16.87 | 37.97 | 72.42 | 1.60 | 1.64 | 2.05 | Rhyolite |
| CI | Coiba Island | CI180408 | 7.441040 | -81.732770 | 50 | Panama slab window | 48.3 | 1.11 | 9.0 | 18.0 | 1.3 | 2.31 | 3.51 | 0.12 | 8.76 | 0.06 | 0.26 | 0.51 | Basalt |
| CL | Calobre | CL180409 | 8.404480 | -80.803750 | 289 | Panama slab window | 50.9 | 3.26 | 7.5 | 29.0 | 8.9 | 6.93 | 9.98 | 0.74 | 19.36 | 0.15 | 0.38 | 3.99 | Rhyolite |
| CV | Casa Valmor | CV180410 | 8.599200 | -80.131620 | 639 | Panama slab window | 34.9 | 3.75 | 7.5 | 14.0 | 8.4 | 21.93 | 0.19 | 0.89 | 15.05 | 0.17 | 2.43 | 0.58 | Rhyolite |
| CW | Cahuita Well | CW180415 | 9.735746 | -82.825737 | 8 | Active volcanic backarc | NA | NA | NA | NA | 2.1 | 40.00 | 0.15 | NA | 45.21 | 0.19 | 0.60 | 0.74 | Andesite |
| CY | Rio Cayuco | CY170214 | 10.287497 | -84.955524 | 184 | Active volcanic arc | 72.0 | 3.15 | 6.3 | 26.7 | 0.6 | 15.44 | 4.42 | 5.24 | 18.30 | 0.81 | 0.97 | 3.41 | Basaltic Andesite |
| CZ | Salitral Carrizal | CZ180409 | 7.714070 | -81.288320 | 60 | Panama slab window | 26.3 | 0.22 | 10.0 | 11.0 | NA | 0.03 | 0.09 | 0.86 | 3.36 | 0.06 | 0.27 | 0.78 | Basalt |
| EP | Espabel | EP170215 | 9.901885 | -85.454327 | 126 | Costa Rica outer forearc | 26.0 | 0.10 | 10.0 | 4.0 | NA | 0.23 | 0.03 | 0.55 | 1.25 | 0.04 | 0.49 | 0.48 | Basalt |
| ER | Rio Blanco Er Resbala | ER180415 | 9.938223 | -83.161331 | 89 | Active volcanic backarc | NA | NA | NA | NA | 6.5 | 12.52 | 0.43 | NA | 7.93 | 0.07 | 0.38 | 1.46 | Picro Basalt |
| ES | Estrada | ES170215 | 9.899005 | -85.453514 | 122 | Costa Rica outer forearc | 27.0 | 0.11 | 9.8 | 48.3 | NA | 1.06 | 0.16 | 1.14 | 2.29 | 0.14 | 0.04 | 0.31 | Basalt |
| ET | Eco Termales | ET170220 | 10.484006 | -84.675853 | 368 | Active volcanic arc | 40.0 | 0.97 | 6.0 | 13.4 | NA | 8.16 | 1.55 | NA | 4.29 | 0.65 | 3.64 | 1.75 | Basaltic Andesite |
| FA | Finca Ande | FA170219 | 10.336843 | -85.069499 | 109 | Active volcanic arc | 55.0 | 2.97 | 5.9 | 10.3 | 4.4 | 20.37 | 6.19 | 14.22 | 25.04 | 1.67 | 1.67 | 2.66 | Basaltic Andesite |
| GE | Gevi | GE140403 | 9.194833 | -83.280806 | 456 | Cordillera Talamanca | 35.8 | 0.45 | 7.8 | 85.0 | NA | 1.08 | 1.16 | 1.22 | 77.71 | 1.61 | 6.94 | 2.03 | Basalt |
| HA | Hattillo | HA180403 | 9.360220 | -83.916640 | 118 | Costa Rica outer forearc | 33.0 | 4.97 | 8.9 | 26.0 | 1.7 | 27.38 | 9.88 | 0.12 | 20.43 | 0.24 | 1.34 | 7.12 | Basalt |
| HN | Hornillas | HN170219 | 10.712822 | -85.177404 | 765 | Active volcanic arc | 87.9 | 7.09 | 1.8 | 57.0 | 6.7 | 0.24 | 12.00 | 1.12 | 0.53 | 0.17 | 0.58 | 1.32 | Andesite |
| LB | Los Bajos | LB180410 | 8.807360 | -79.790610 | 26 | Panama slab window | 34.8 | 25.35 | NA | 70.0 | NA | NA | NA | 17.74 | 135.06 | 2.69 | 4.50 | 0.42 | Rhyolite |
| LE | Las Estrella | LE180416 | 10.427103 | -84.368543 | 153 | Active volcanic arc | NA | NA | NA | NA | NA | 17.56 | 0.38 | NA | 11.37 | 0.62 | 2.40 | 14.06 | Basalt |
| LH | Los Pozos Termales (hot) | LH180406 | 8.870950 | -82.689900 | 1676 | Cordillera Talamanca | 55.4 | 8.43 | 6.7 | 12.0 | 7.6 | 37.97 | 2.53 | 33.54 | 31.02 | 1.26 | 0.65 | 0.43 | Andesite |
| LP | Los Pozos Termales (warm) | LP180406 | 8.869660 | -82.692820 | 1651 | Cordillera Talamanca | 39.1 | 5.88 | 6.5 | 32.0 | 7.0 | 30.45 | 1.74 | 31.33 | 30.18 | 1.49 | 0.94 | 1.36 | Andesite |
| LW | Laurel | LW180405 | 8.441190 | -82.904870 | 32 | Costa Rica outer forearc | 31.5 | 0.55 | 7.1 | 3.5 | 1.2 | 0.15 | 0.01 | 7.11 | 1.97 | 0.26 | 0.76 | 0.48 | Andesite |
| MC | Montecarlo - Bernardino | MC180404 | 9.343910 | -83.595650 | 812 | Cordillera Talamanca | 31.8 | 2.33 | 9.6 | 23.0 | 2.4 | 20.06 | 4.94 | 0.04 | 13.81 | 0.09 | 0.06 | 3.54 | Basalt |
| MT | Mouse Trap | MT170219 | 10.595774 | -85.238451 | 166 | Active volcanic arc | 59.0 | 3.34 | 6.3 | 12.4 | 3.6 | 34.42 | 0.14 | 21.70 | 34.24 | 1.06 | 0.13 | 0.70 | Rhyolite |
| PF | Pompilos finca | PF170222 | 10.518466 | -84.115180 | 53 | Active volcanic backarc | 28.0 | 1.96 | 5.8 | 4.3 | 1.7 | 17.50 | 0.18 | 23.72 | 12.38 | 1.10 | 6.31 | 0.81 | Andesite |
| PG | Poas Volcano Laguna | PG170224 | 10.188962 | -84.227388 | 1632 | Active volcanic arc | NA | NA | NA | NA | 6.5 | 0.19 | 0.05 | NA | 0.11 | 0.03 | 0.16 | 0.20 | Andesite |
| PL | Poas Volcano lake | PL170224 | 10.196777 | -84.229892 | 2334 | Active volcanic arc | 37.0 | 65.75 | 0.9 | 46.6 | 6.5 | 109.72 | 99.22 | 0.86 | 3.36 | 1.60 | 4.85 | 18.08 | Andesite |
| PS | Playa Sandalo | PS180405 | 8.575540 | -83.364160 | 2 | Costa Rica outer forearc | 33.0 | 69.20 | 8.2 | 122.0 | 3.3 | NA | NA | 3.18 | 345.81 | 7.06 | 34.79 | 7.12 | Andesite |
| PX | Praxair well 24 | PX180416 | 10.485523 | -84.113229 | 68 | Active volcanic backarc | NA | NA | NA | NA | 7.8 | 16.78 | 0.57 | NA | 2.19 | 0.18 | 2.89 | 11.15 | Basalt |
| QH1 | Quepos Hot Springs 1 | QH170213_1 | 9.561710 | -84.123251 | 298 | Costa Rica outer forearc | 48.0 | 1.39 | 8.7 | 47.3 | 3.1 | 25.21 | 2.06 | 0.10 | 17.02 | 0.23 | 0.02 | 4.86 | Basalt |
| QH2 | Quepos Hot Springs 2 | QH170213_2 | 9.561710 | -84.123251 | 300 | Costa Rica outer forearc | 36.0 | 1.39 | 8.7 | 47.3 | 3.1 | 14.64 | 0.95 | 0.11 | 10.70 | 0.14 | 0.02 | 2.20 | Basalt |
| QN | Quebrada naranja | QN170220 | 10.495573 | -84.696714 | 429 | Active volcanic arc | 23.0 | 0.10 | 5.6 | 84.0 | 4.9 | 0.31 | 0.03 | 2.81 | 0.46 | 0.12 | 0.29 | 0.73 | Andesite |
| RC | Ujarassa | RC180404 | 9.302830 | -83.297820 | 943 | Cordillera Talamanca | 60.0 | 2.83 | 7.7 | 45.0 | NA | NA | NA | 1.07 | 15.90 | 0.20 | 0.21 | 1.81 | Basalt |
| RR | Rockslide | RR180407 | 8.635910 | -82.223690 | 792 | Cordillera Talamanca | 41.3 | 2.51 | NA | 73.0 | NA | NA | NA | 0.32 | 8.96 | 0.11 | 0.09 | 5.84 | Andesite |
| RS | Ranchero el Salitral | RS170216 | 10.232331 | -85.531602 | 82 | Costa Rica outer forearc | 29.0 | 0.13 | 10.0 | 5.6 | 2.7 | 0.65 | 0.07 | 0.25 | 1.60 | 0.02 | 0.01 | 0.08 | Rhyolite |
| RV | Recreo Verde | RV170221 | 10.321576 | -84.243686 | 557 | Active volcanic arc | 42.7 | 62.86 | 6.2 | 1.8 | 6.7 | 14.60 | 4.48 | 19.73 | 14.31 | 2.58 | 13.54 | 0.40 | Basaltic Andesite |
| SC | El Salao Campollano | SC180411 | 8.157550 | -81.130970 | 136 | Panama slab window | 29.9 | 56.21 | 6.5 | 30.0 | 7.6 | NA | NA | 58.45 | 255.19 | 6.82 | 4.43 | 10.98 | Basalt |
| SI | El Sitio | SI170217 | 10.301239 | -85.610549 | 36 | Costa Rica outer forearc | 36.0 | 1.82 | 9.8 | 36.2 | 0.4 | 0.64 | 0.03 | 2.24 | 1.11 | 0.03 | 0.02 | 0.15 | Rhyolite |
| SL | Santa Lucia | SL170214 | 10.290599 | -84.972435 | 165 | Active volcanic arc | 57.0 | 1.46 | 6.1 | 22.0 | 3.8 | 3.52 | 5.02 | 5.69 | 11.83 | 0.54 | 1.07 | 2.74 | Basaltic Andesite |
| ST | Santa Teresa | ST170223 | 10.002942 | -83.827507 | 2209 | Active volcanic arc | 55.8 | 2.98 | 4.5 | 4.7 | NA | NA | NA | 9.94 | NA | NA | NA | NA | Andesite |
| TC | El Tucano bubbling site | TC170221 | 10.366486 | -84.381208 | 553 | Active volcanic arc | 60.0 | 1.84 | 6.3 | 18.7 | 6.6 | 18.53 | 0.06 | 13.57 | 12.96 | 1.31 | 2.29 | 1.06 | Basaltic Andesite |
| VC | Blue River Volcancito | VC170218 | 10.897847 | -85.326461 | 436 | Active volcanic arc | 59.8 | 7.19 | 5.0 | 17.1 | 6.9 | 27.07 | 4.65 | 6.13 | 8.55 | 2.77 | 10.72 | 7.38 | Andesite |
| XF | Praxair well 19 | XF180416 | 10.485523 | -84.113229 | 74 | Active volcanic backarc | NA | NA | NA | NA | 7.6 | NA | NA | NA | 15.53 | 0.67 | 3.59 | 4.67 | Basalt |
| YR | Yheri | YR180404 | 9.194920 | -83.280590 | 469 | Cordillera Talamanca | 26.0 | 4.59 | 8.9 | 10.0 | 2.9 | NA | NA | 0.06 | 28.90 | 0.28 | 0.28 | 7.51 | Basalt |

**Supplementary Table S2. Envfit results against nMDS1, nMDS2 and nMDS3.** MAP = Mean annual Precipitation, Dsub = Distance from subduction, DOT = Distance from oceanic transform, DHS = Distance from hot spots, DV = Distance from volcanoes, DEQ = Distance from significant earthquakes, DCS = Continental shelf, SDepth = Slab depth, SDip = Slab dip angle, ConvR = Convergence rate and ConvObl = Convergence obliquity. One star (*); p-value less than 0.01, two stars (**) p-value is less than 0.001. Only variables with a p-value of less than 0.01 and a r^2^ > 0.32 (for N=72) are marked in bold and considered statistically significant.

|  | **Variable** | **NMDS1** | **NMDS2** | **r^2^** | **Pr(>r)** |  | **NMDS1** | **NMDS3** | **r^2^** | **Pr(>r)** |  |
| --- | --- | --- | --- | --- | --- | --- | --- | --- | --- | --- | --- |
| **Location** | latitude | 0.2839 | 0.95885 | 0.1588 | 0.84167 |  | 0.06719 | -0.99774 | 0.4172 | 0.591667 |  |
|  | longitude | -0.32717 | -0.94497 | 0.67 | 0.275 |  | -0.95914 | -0.28292 | 0.317 | 0.733333 |  |
|  | Altitude | -0.35764 | -0.93386 | 0.3141 | 0.76667 |  | -0.25508 | -0.96692 | 0.7729 | 0.233333 |  |
|  | MAP | -0.53001 | -0.84799 | 0.7772 | 0.21667 |  | -0.85999 | -0.51031 | 0.7377 | 0.26667 |  |
|  |  |  |  |  |  |  |  |  |  |  |  |
|  | **Variable** | **NMDS1** | **NMDS2** | **r^2^** | **Pr(>r)** |  | **NMDS1** | **NMDS3** | **r^2^** | **Pr(>r)** |  |
| **Physico-Chemical** | Temp | 0.50604 | -0.86251 | 0.913 | 0.18333 |  | 0.95382 | 0.30038 | 0.9012 | 0.1 |  |
|  | Salinity | 0.88148 | 0.47221 | 0.9712 | 0.09167 |  | 0.99138 | 0.13101 | 0.9671 | 0.1 |  |
|  | pH | 0.12583 | -0.99205 | 0.9739 | 0.09167 |  | 0.9998 | 0.02003 | 0.8471 | 0.216667 |  |
|  | DO | -0.23995 | 0.97079 | 0.9451 | 0.125 |  | -0.72766 | 0.68594 | 0.9724 | 0.033333 |  |
|  | TOC | -0.20459 | -0.97885 | 0.6015 | 0.35833 |  | -0.01544 | -0.99988 | 0.4563 | 0.525 |  |
|  | DIC | -0.11408 | -0.99347 | 0.5904 | 0.43333 |  | 0.48306 | -0.87559 | 0.3124 | 0.733333 |  |
|  | CO2 | 0.23672 | -0.97158 | 0.5994 | 0.38333 |  | 0.52539 | 0.85086 | 0.7969 | 0.191667 |  |
|  | **Rc/Ra** | -0.29792 | -0.95459 | 0.209 | 0.825 |  | -0.15041 | -0.98862 | **0.9999** | **0.008333** | ****** |
|  | Cl- | 0.32508 | -0.94569 | 0.716 | 0.31667 |  | 0.67862 | -0.73449 | 0.7779 | 0.241667 |  |
|  | SO42- | 0.32475 | 0.9458 | 0.8157 | 0.23333 |  | 0.76162 | 0.64802 | 0.4133 | 0.45 |  |
|  | Na+ | 0.07386 | -0.99727 | 0.5132 | 0.50833 |  | 0.71114 | 0.70305 | 0.4898 | 0.516667 |  |
|  | K+ | 0.70052 | 0.71363 | 0.4022 | 0.61667 |  | 0.28807 | -0.95761 | 0.832 | 0.183333 |  |
|  | Mg2+ | 0.35819 | 0.93365 | 0.255 | 0.675 |  | 0.17087 | -0.98529 | 0.5739 | 0.45 |  |
|  | Ca2+ | 0.32844 | 0.94452 | 0.6632 | 0.38333 |  | 0.70802 | -0.70619 | 0.3536 | 0.683333 |  |
|  |  |  |  |  |  |  |  |  |  |  |  |
|  | **Variable** | **NMDS1** | **NMDS2** | **r^2^** | **Pr(>r)** |  | **NMDS1** | **NMDS3** | **r^2^** | **Pr(>r)** |  |
| **Geophysical** | **DPM** | -0.92119 | 0.38912 | 0.0849 | 0.039 |  | -0.38872 | -0.92136 | **0.385** | **0.001** | ******* |
|  | **CT** | 0.99362 | 0.11279 | 0.0563 | 0.121 |  | 0.35252 | 0.93581 | **0.3455** | **0.001** | ******* |
|  | SedThic_On | -0.47271 | -0.88122 | 0.002 | 0.914 |  | -0.19124 | -0.98154 | 0.0115 | 0.667 | . |
|  | DistCoast | -0.58735 | 0.80933 | 0.0858 | 0.058 | . | -0.96327 | 0.26853 | 0.0373 | 0.265 |  |
|  | DSub | -0.0649 | 0.99789 | 0.201 | 0.001 | *** | -0.10618 | -0.99435 | 0.0736 | 0.065 | . |
|  | **DMOR** | 0.97889 | 0.20436 | 0.0758 | 0.066 |  | 0.41753 | 0.90866 | **0.3297** | **0.001** | ******* |
|  | DOT | 0.90511 | 0.42518 | 0.0956 | 0.029 |  | 0.52753 | 0.84954 | 0.2389 | 0.001 | *** |
|  | DHS | -0.32829 | 0.94458 | 0.2397 | 0.001 | *** | -0.34206 | -0.93968 | 0.2172 | 0.003 | ** |
|  | DV | -0.32343 | -0.94625 | 0.1671 | 0.006 | ** | -0.75568 | -0.65494 | 0.0348 | 0.278 |  |
|  | **DMUSGS** | 0.87409 | -0.48577 | 0.1024 | 0.017 |  | 0.39609 | 0.91821 | **0.4123** | **0.001** | ******* |
|  | DEQ | -0.4429 | 0.89657 | 0.1932 | 0.001 | *** | -0.41186 | -0.91125 | 0.2176 | 0.002 | ** |
|  | HF | -0.69084 | -0.72301 | 0.0697 | 0.07 |  | -0.78888 | -0.61455 | 0.0552 | 0.123 |  |
|  | PV | -0.64153 | -0.7671 | 0.0857 | 0.039 |  | -0.56331 | -0.82624 | 0.1066 | 0.025 |  |
|  | DCS | -0.3991 | 0.91691 | 0.1047 | 0.015 | . | -0.24496 | -0.96953 | 0.2637 | 0.001 | *** |
|  | SDepth | 0.2265 | -0.97401 | 0.0042 | 0.871 |  | 0.06704 | -0.99775 | 0.046 | 0.205 |  |
|  | SDip | 0.9033 | 0.42901 | 0.0123 | 0.657 |  | 0.30748 | 0.95156 | 0.0846 | 0.037 |  |
|  | ConvR | 0.26122 | -0.96528 | 0.107 | 0.021 |  | 0.20437 | 0.97889 | 0.1696 | 0.001 | *** |
|  | ConvObl | -0.50669 | 0.86213 | 0.0377 | 0.249 |  | -0.24096 | -0.97053 | 0.1533 | 0.003 | ** |
|  | **ConvAge** | -0.89875 | 0.43847 | 0.0987 | 0.019 |  | -0.41248 | -0.91097 | **0.3849** | **0.001** | ******* |
|  |  |  |  |  |  |  |  |  |  |  |  |
|  | **Variable** | **NMDS1** | **NMDS2** | **r^2^** | **Pr(>r)** |  | **NMDS1** | **NMDS3** | **r^2^** | **Pr(>r)** |  |
| **Rock major** | sio2_d | -0.12496 | 0.99216 | 0.0832 | 0.053 |  | -0.28935 | -0.95722 | 0.0056 | 0.826 |  |
|  | tio2_d | -0.33172 | -0.94338 | 0.103 | 0.033 |  | -0.93815 | -0.34623 | 0.0193 | 0.515 |  |
|  | al2o3_d | 0.27741 | 0.96075 | 0.0807 | 0.05 |  | 0.27437 | 0.96162 | 0.0807 | 0.057 |  |
|  | FEOt_d | 0.39821 | -0.91729 | 0.1165 | 0.016 | . | 0.86367 | 0.50405 | 0.0245 | 0.456 |  |
|  | mno_d | 0.1395 | -0.99022 | 0.0111 | 0.671 |  | 0.10269 | 0.99471 | 0.0052 | 0.859 |  |
|  | mgo_d | 0.22973 | -0.97325 | 0.1651 | 0.007 | ** | 0.68906 | 0.7247 | 0.0138 | 0.63 |  |
|  | cao_d | 0.33713 | -0.94146 | 0.1552 | 0.004 | ** | 0.60029 | 0.79978 | 0.0396 | 0.263 |  |
|  | **na2o_d** | -0.39293 | 0.91957 | **0.3285** | **0.001** | ******* | -0.45298 | -0.89152 | 0.1863 | 0.002 | ** |
|  | k2o_d | -0.7348 | 0.67829 | 0.0784 | 0.064 |  | -0.72614 | -0.68755 | 0.0746 | 0.084 | . |
|  | p2o5_d | -0.99194 | -0.1267 | 0.0547 | 0.146 |  | -0.99925 | -0.03871 | 0.0541 | 0.172 |  |
|  |  |  |  |  |  |  |  |  |  |  |  |
|  | **Variable** | **NMDS1** | **NMDS2** | **r^2^** | **Pr(>r)** |  | **NMDS1** | **NMDS3** | **r^2^** | **Pr(>r)** |  |
| **Rock trace** | Cu | 0.70491 | 0.7093 | 0.0618 | 0.667 |  | 0.14998 | -0.98869 | 0.2789 | 0.109 |  |
|  | Co | 0.78381 | 0.621 | 0.079 | 0.602 |  | 0.20264 | -0.97925 | 0.3124 | 0.07 |  |
|  | Mo | 0.82472 | 0.56554 | 0.0867 | 0.57 |  | 0.22382 | -0.97463 | 0.3282 | 0.064 |  |
|  | **Ni** | -0.61896 | 0.78542 | 0.4453 | 0.029 |  | -0.56689 | 0.82379 | **0.6356** | **0.002** | ****** |
|  | V | -0.94336 | -0.33177 | 0.1208 | 0.44 |  | -0.29391 | 0.95583 | 0.3869 | 0.044 |  |
|  | Zn | -0.64882 | -0.76094 | 0.0725 | 0.637 |  | -0.16609 | 0.98611 | 0.2601 | 0.112 |  |
|  | Ba | -0.90248 | 0.43073 | 0.1151 | 0.453 |  | -0.32165 | 0.94686 | 0.3343 | 0.067 |  |
|  | Ce | -0.78144 | 0.62398 | 0.2354 | 0.18 | . | -0.43221 | 0.90178 | 0.4799 | 0.011 | . |
|  | Cr | 0.19926 | -0.97995 | 0.0936 | 0.478 |  | 0.73015 | 0.68328 | 0.077 | 0.572 |  |
|  | **Dy** | 0.97266 | -0.23224 | 0.2432 | 0.162 |  | 0.44295 | -0.89655 | **0.5209** | **0.008** | ****** |
|  | Er | -0.35206 | -0.93598 | 0.0612 | 0.698 |  | 0.05518 | 0.99848 | 0.1757 | 0.263 |  |
|  | Eu | -0.87308 | -0.48758 | 0.0804 | 0.593 |  | -0.21787 | 0.97598 | 0.3288 | 0.071 |  |
|  | Gd | -0.55118 | -0.83439 | 0.0317 | 0.803 |  | -0.03687 | 0.99932 | 0.1991 | 0.228 |  |
|  | Ho | -0.18934 | -0.98191 | 0.0908 | 0.563 |  | 0.24048 | 0.97065 | 0.1317 | 0.382 |  |
|  | La | -0.48244 | 0.87593 | 0.3541 | 0.08 |  | -0.57862 | 0.8156 | 0.4491 | 0.025 |  |
|  | Lu | 0.80468 | 0.5937 | 0.0828 | 0.584 |  | 0.21358 | -0.97693 | 0.3204 | 0.067 |  |
|  | Nb | -0.01062 | 0.99994 | 0.2385 | 0.186 |  | -0.85877 | -0.51235 | 0.0792 | 0.594 |  |
|  | Nd | -0.93353 | 0.35851 | 0.1756 | 0.291 |  | -0.3682 | 0.92975 | 0.4486 | 0.019 | . |
|  | Pb | 0.80573 | 0.59229 | 0.0832 | 0.582 |  | 0.2144 | -0.97675 | 0.3208 | 0.067 |  |
|  | Pr | -0.00554 | 0.99998 | 0.2014 | 0.243 |  | -0.92979 | -0.36809 | 0.0585 | 0.68 |  |
|  | Rb | -0.5489 | 0.83589 | 0.0984 | 0.485 |  | -0.36735 | 0.93008 | 0.2083 | 0.212 |  |
|  | Sm | -0.86978 | 0.49344 | 0.1235 | 0.419 |  | -0.33551 | 0.94204 | 0.3391 | 0.066 |  |
|  | Sr | 0.03572 | 0.99936 | 0.1807 | 0.293 |  | -0.55241 | -0.83357 | 0.1079 | 0.468 |  |
|  | Ta | 0.76561 | 0.6433 | 0.0775 | 0.609 |  | 0.19672 | -0.98046 | 0.3065 | 0.076 |  |
|  | Tb | -0.14256 | -0.98979 | 0.081 | 0.59 |  | 0.29141 | 0.9566 | 0.1238 | 0.405 |  |
|  | Th | 0.07484 | 0.9972 | 0.1176 | 0.469 |  | -0.81833 | -0.57475 | 0.0235 | 0.87 |  |
|  | U | 0.39879 | 0.91704 | 0.0684 | 0.688 |  | 0.03642 | -0.99934 | 0.1209 | 0.415 |  |
|  | **Y** | 0.63262 | -0.77446 | 0.5418 | 0.011 | . | 0.6158 | -0.7879 | **0.7192** | **0.001** | ******* |
|  | Yb | 0.93756 | 0.34783 | 0.1167 | 0.455 |  | 0.28633 | -0.95813 | 0.3823 | 0.046 |  |
|  | Zr | -0.25067 | 0.96807 | 0.1661 | 0.288 |  | -0.60514 | 0.79612 | 0.1515 | 0.322 |  |

## Supplementary Figures

**Supplementary Fig. S1. Changing in volatile sources in the sample dataset as shown by the ^3^He/^4^He ratio expressed as Rc/Ra. A** ^3^He/^4^He and distance from subduction (in km) across the sampled area; **B** Regression analysis depicting positive relationship between distance from subduction and ^3^He/^4^He ratio (Rc/Ra) (r = 0.59, p < 0.001, n = 34). Imagery reproduced from the GEBCO_2022 Grid, GEBCO Compilation Group (2022) GEBCO 2022 Grid (doi:10.5285/e0f0bb80-ab44-2739-e053-6c86abc0289c).

**
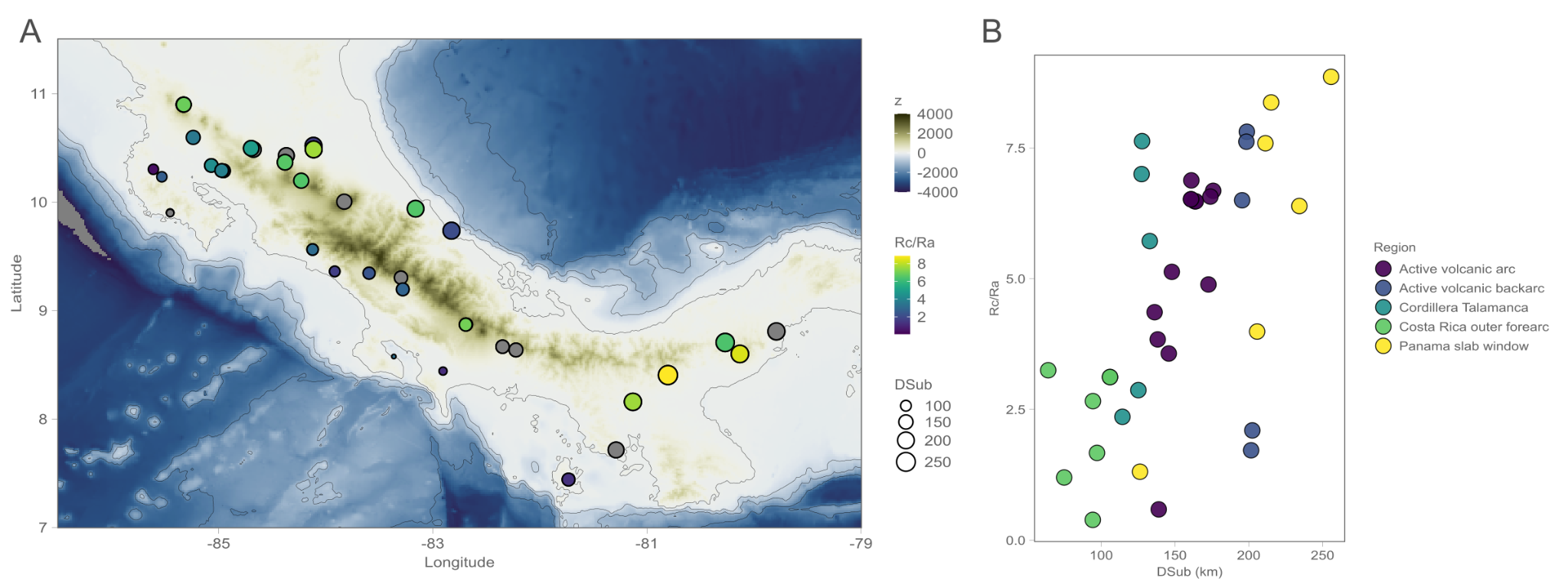
**

**Supplementary Fig. S2. Changes in Temperature (A), pH (B) and ^3^He/^4^He (C) among the different sampled regions.**  One star (*); p-value less than 0.05, two stars (**) p-value is less than 0.01 and if p-value is less than 0.001, it is flagged with three stars (***).

**
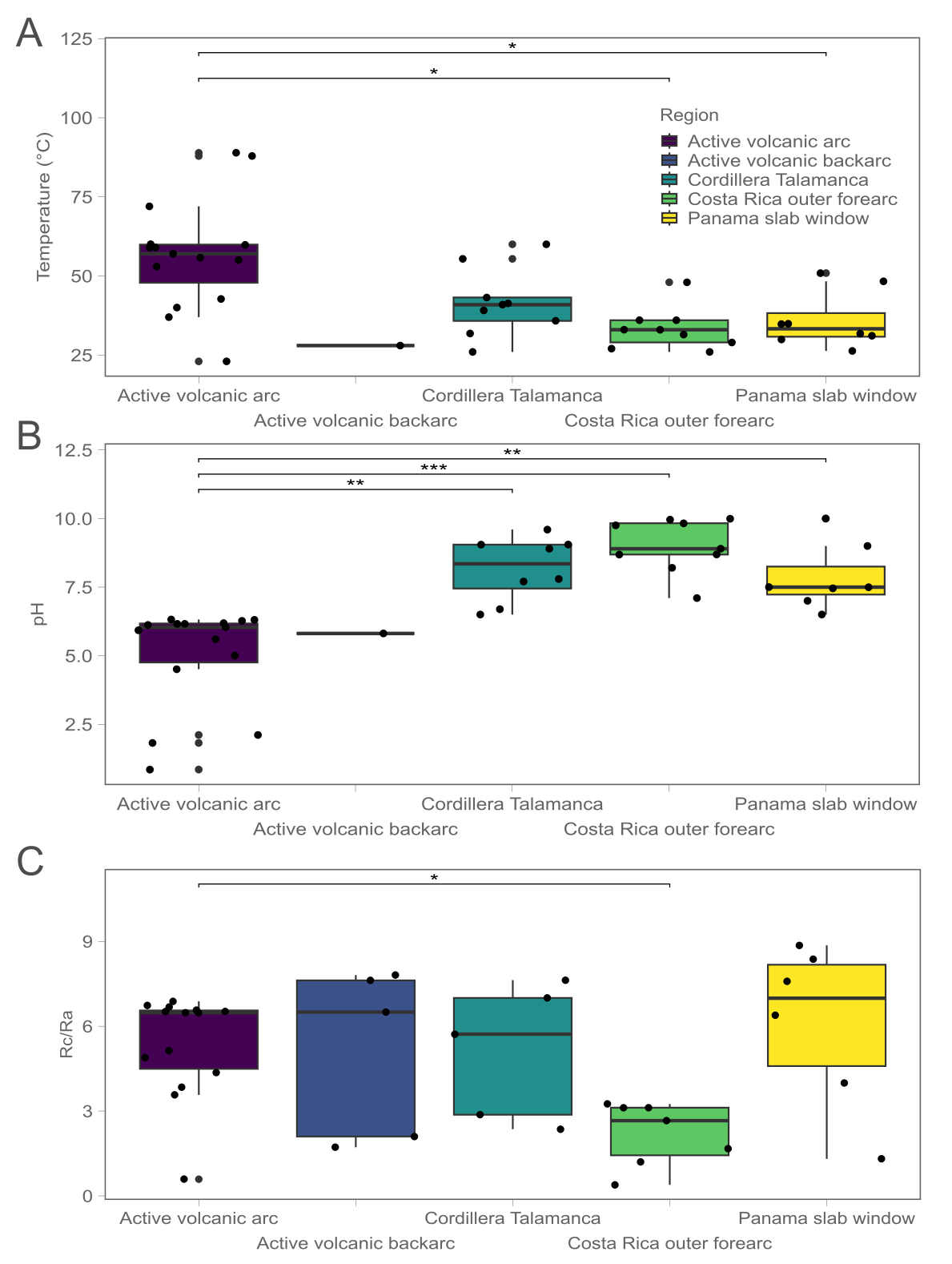
**

**Supplementary Fig. S3. Ternary K-Na-Mg diagram after Giggenbach^3^ for evaluating the equilibrium temperature of representative fluid samples.**

**
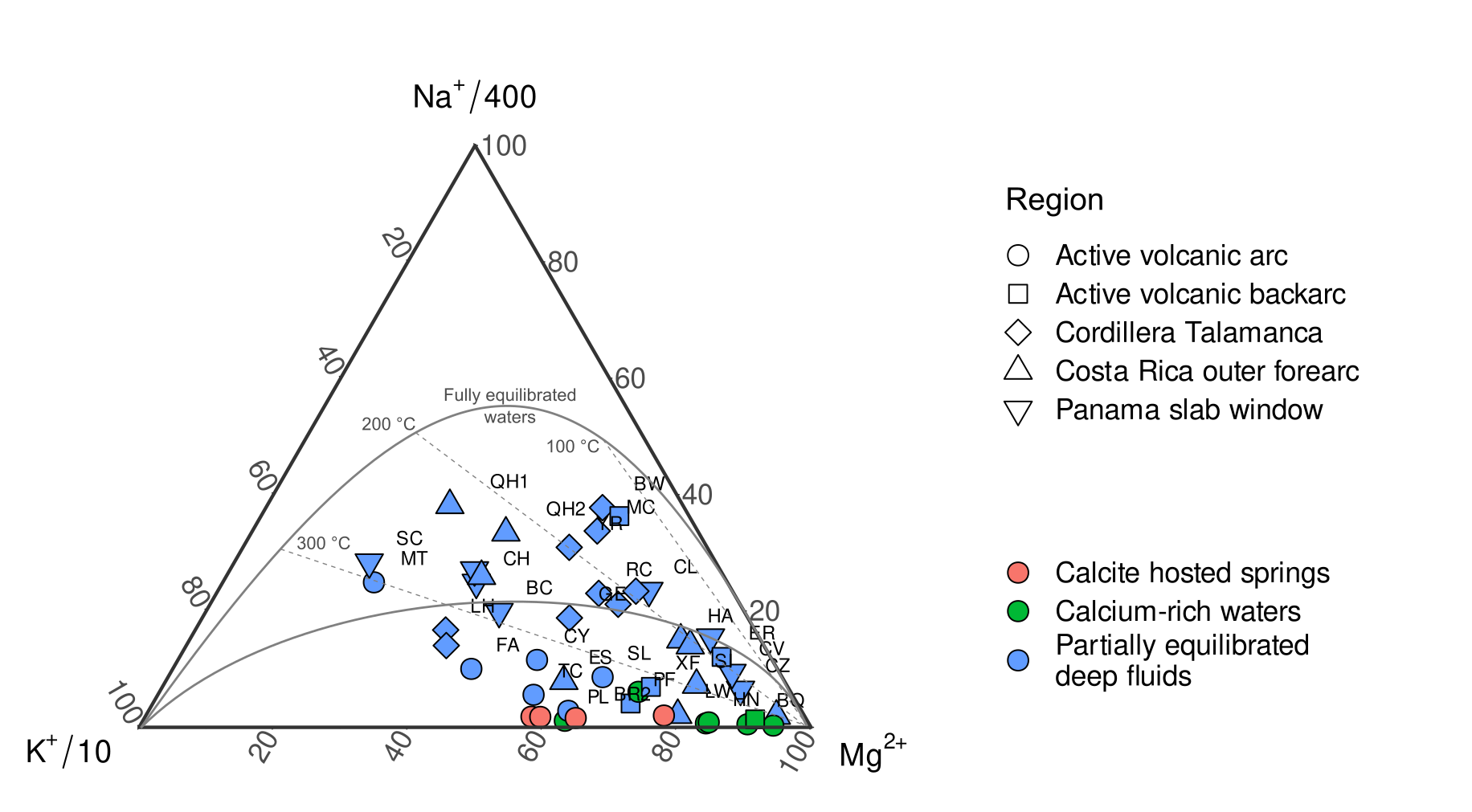
**

**Supplementary Fig. S4. Matrix of correlation (Pearson correlation) among the environmental, geophysical, geochemical (major ions and trace element) variables showing collinearity among some of the investigated parameters. The dendrogram on the left side of the heatmap shows clustering based on similarity of distributions of that geochemical variable across sites.**

**
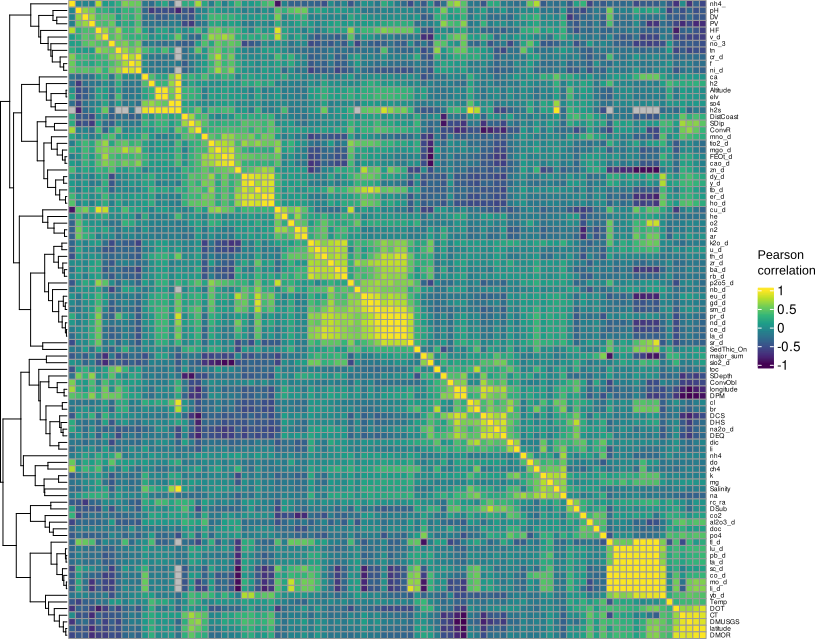
**

**Supplementary Fig. S5. Shannon diversity metrics across geographical regions.**

**
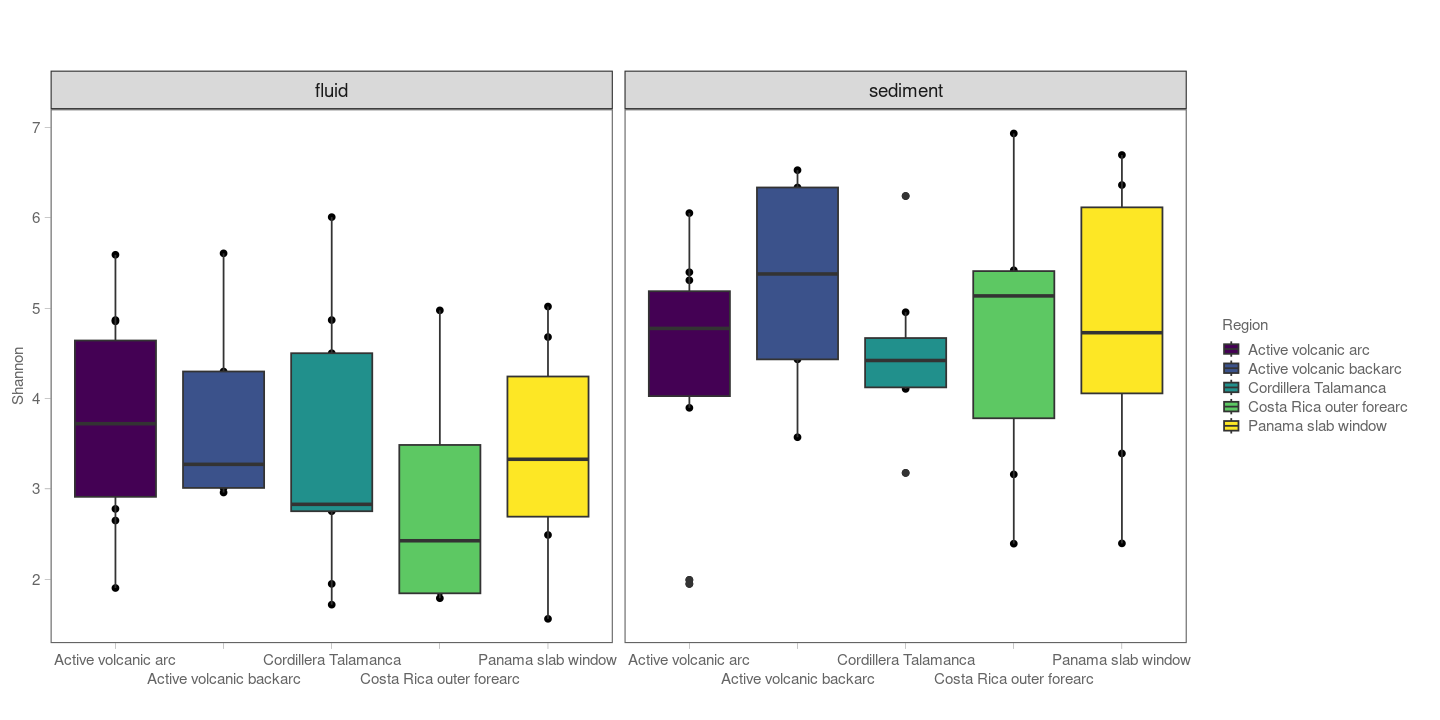
**

**Supplementary Fig. S6. Non-metric multidimensional scaling (nMDS) plot based on the Jensen-Shannon Divergence colored by region with individual sites labeled.**


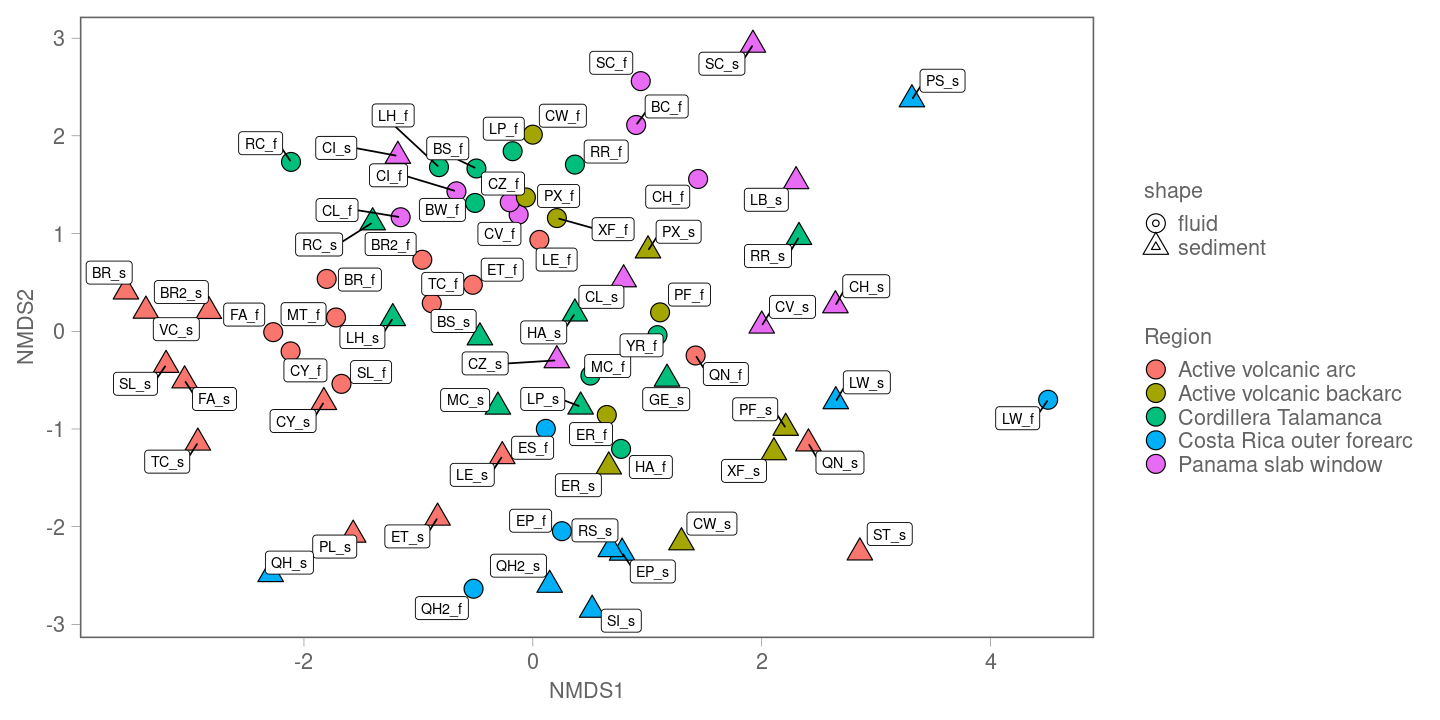


**Supplementary Fig. S7. 3D view of the non-metric multidimensional scaling (nMDS) plot based on the Jensen-Shannon Divergence and colored by region.**

**
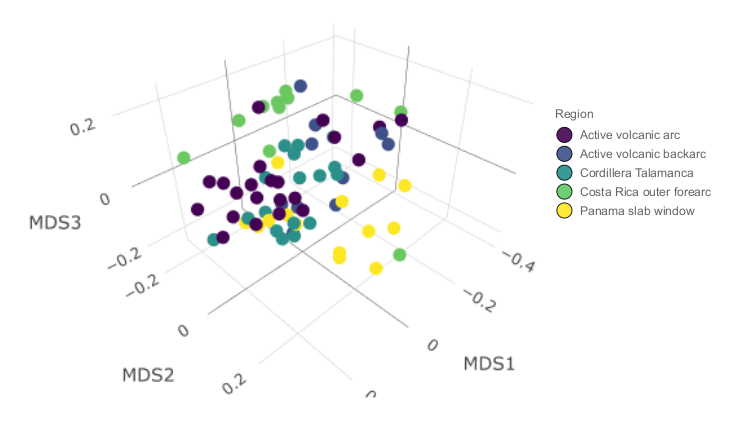
**

**Supplementary Fig. S8. Non-metric multidimensional scaling (nMDS) plot based on the weighted Jensen-Shannon Divergence coloured according to region (ellipses represent the 95% confidence interval of the group scoring).**

**
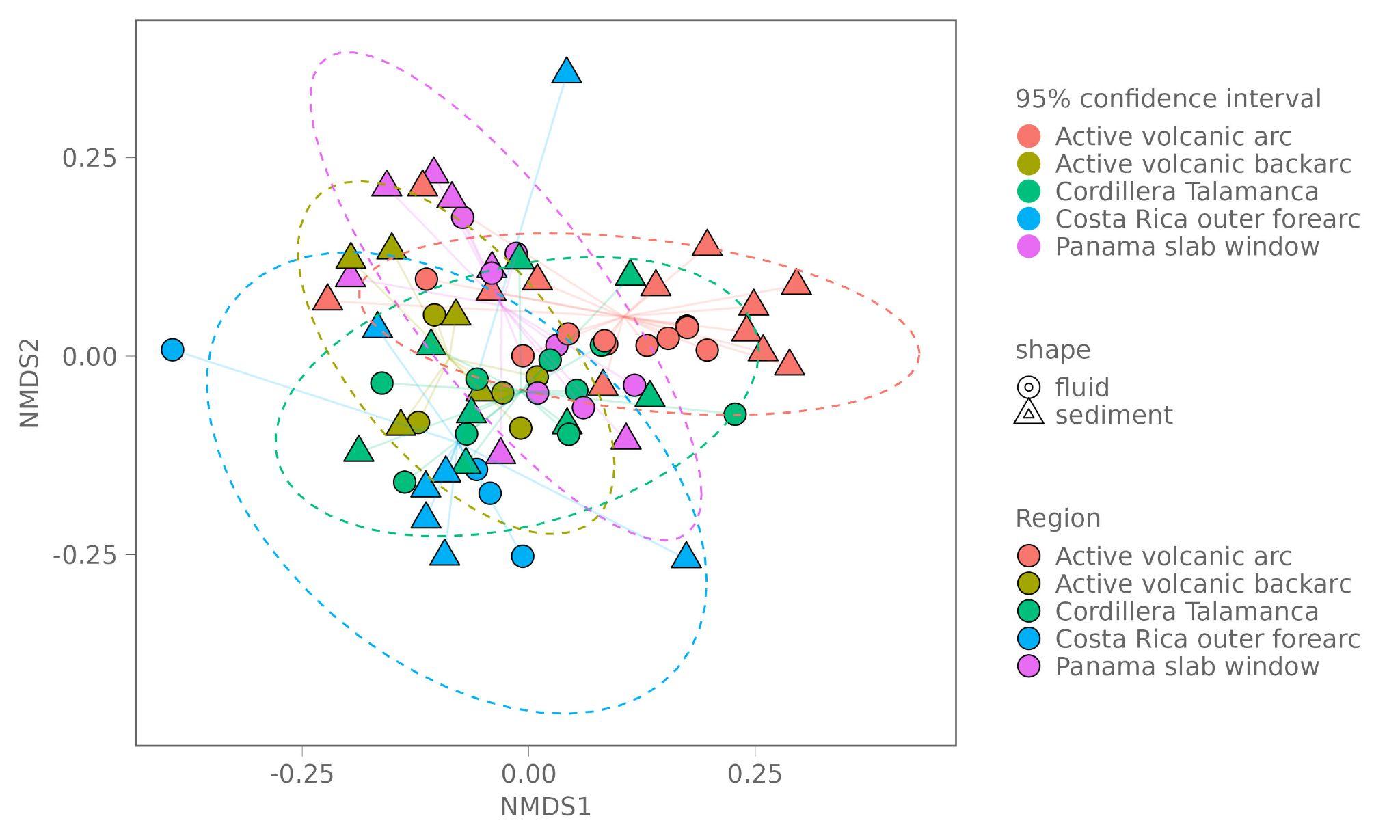
**

**Supplementary Fig. S9. Correlation between the Jensen-Shannon Divergence matrix and the pairwise geographical distance matrix to test for spatial autocorrelation.**

**
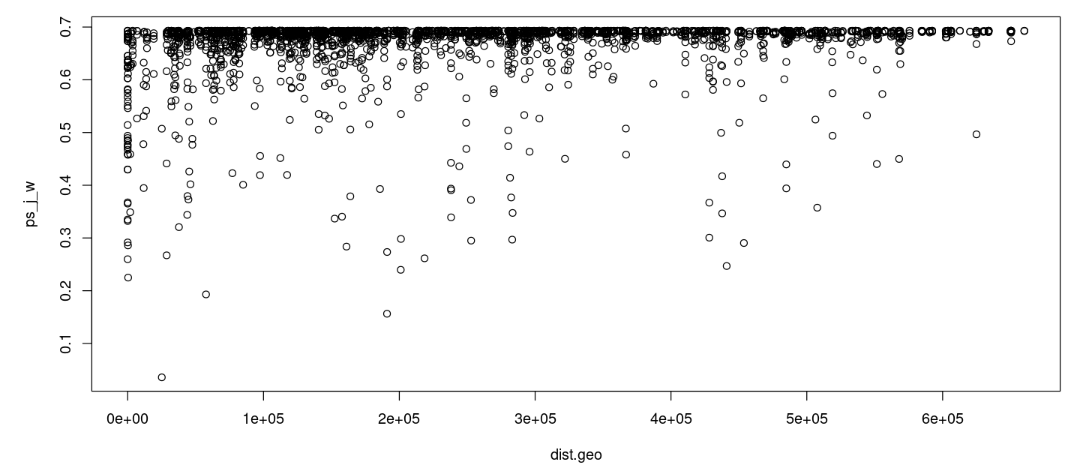
**

**Supplementary Fig. S10. Comparison of noble gases demonstrating little air or air-saturated water inputs to samples from this study.** Distinguished from surficial groundwater from drinking wells that more closely reflects saturation with air.

**
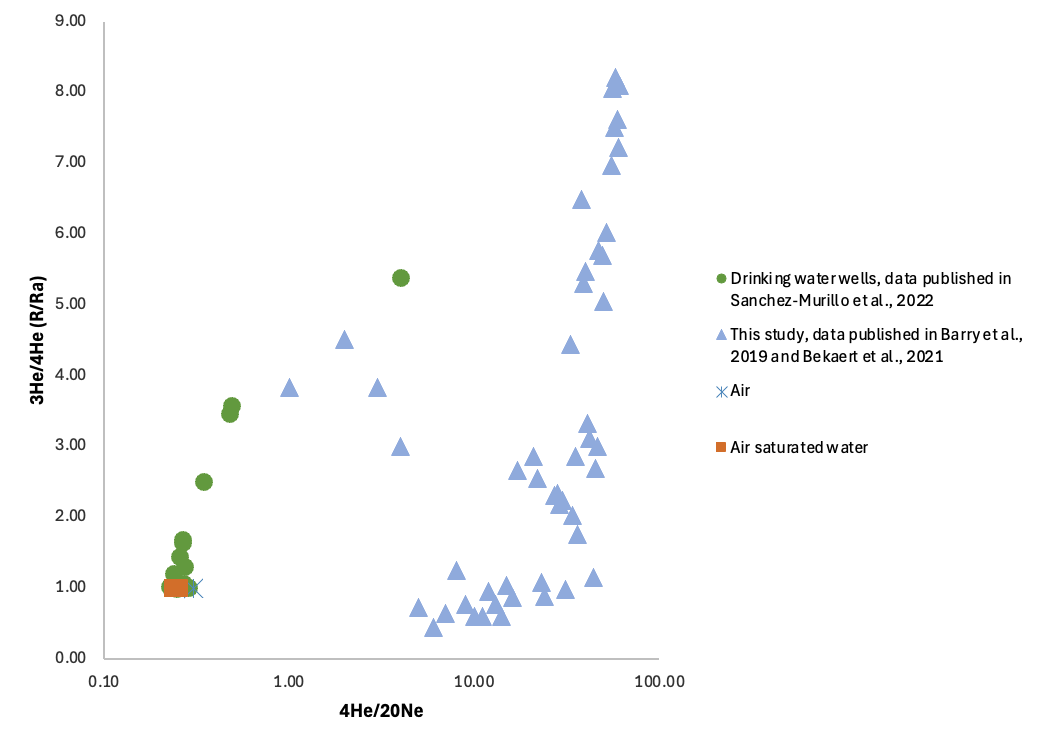
**

**Supplementary Fig. S11. Non-metric multidimensional scaling (nMDS) plot on the Jensen-Shannon Divergence distance between samples (stress = 0.17).** Arrows show significant (p < 0.05) envfit results grouped by type of variables.


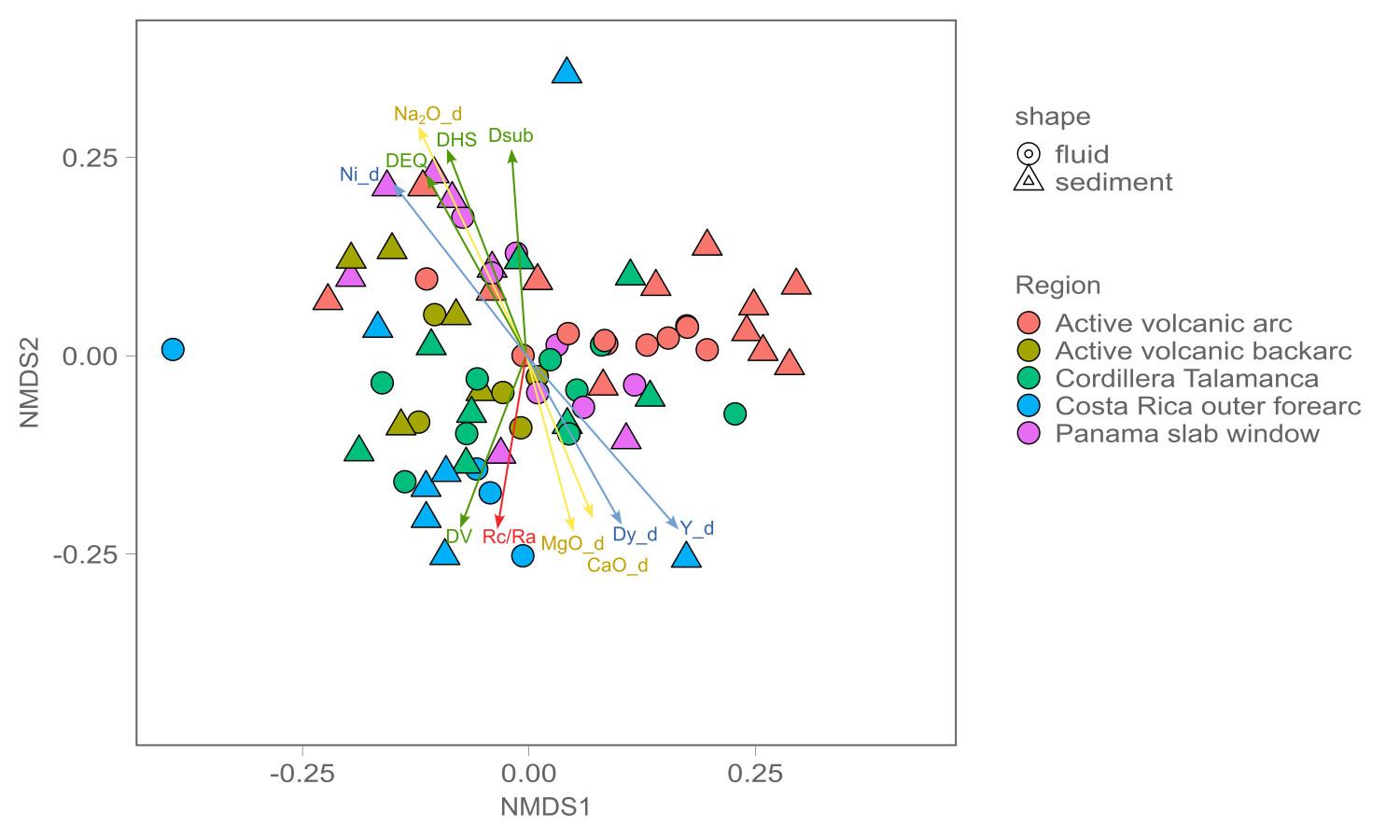

Supplement: S1 File — (DOCX) [file pone.0308756.s001.docx]
